# Knowledge Hiding: Current Research Status and Future Research Directions

**DOI:** 10.3389/fpsyg.2021.748237

**Published:** 2021-10-29

**Authors:** Peixu He, Cuiling Jiang, Zhixing Xu, Chuangang Shen

**Affiliations:** ^1^Business School, Huaqiao University, Quanzhou, China; ^2^Department of Management, Kedge Business School, Talence, France; ^3^Business School, Beijing Normal University, Beijing, China

**Keywords:** knowledge hiding, systematic literature review, future research directions, content analysis, bibliometric analysis, descriptive analysis

## Abstract

This article provides a review of scientific articles addressing the topic of knowledge hiding in organizations. Based on a descriptive analysis, bibliometric analysis, and content analysis of a sample of 81 articles published in the academic journals in the Web of Science from 2012 to 2020, we identify the main areas and current dynamics of knowledge hiding research. Our results show that the central research themes of knowledge hiding include five clusters: concept and dimensions, antecedents, consequences, theories, and influence mechanisms. Based on our findings, we suggest future research should further develop the concept and dimensions of knowledge hiding; probe deeper into the consequences of knowledge hiding; explore multilateral, cross-level, and collective knowledge hiding; employ innovative theoretical perspectives and research methods to study knowledge hiding; and address how cultural and other contextual factors may shape the knowledge hiding behavior.

## Introduction

Knowledge management plays a crucial role in each organization, which can affect the firms' and employees' performance. However, due to the practice of “knowledge hiding,” it is often challenging to achieve satisfactory results in knowledge management (Connelly and Kelloway, 2003). Previous research has pointed out that employees are not willing to share knowledge, due to reasons such as protection and control of knowledge ownership, expertise dominance, and defensive awareness (Huo et al., 2016). About 50% of employees have the intention to withhold, mislead, or conceal knowledge that has been requested by another person (Peng, 2013). This behavior of deliberately not providing the required knowledge to colleagues when requested is called “knowledge hiding” (Connelly et al., 2012), which has become an independent concept that is different from the opposite side of knowledge sharing (Zhao et al., 2019).

Obviously, knowledge hiding is very likely to reduce the efficiency of knowledge exchange among members, hinder the generation of new ideas/thoughts, or even destroy trust (Connelly et al., 2012), increasing the risk of knowledge loss and inhibiting the creativity of individuals and teams (Cerne et al., 2014; Bogilović et al., 2017). Along this vein, it makes sense to solve the dilemma of insufficient knowledge sharing through the elimination of knowledge hiding, facilitating knowledge conversion within organizations. As a result, based on a descriptive analysis, bibliometric analysis, and content analysis, we conduct an in-depth analysis of knowledge hiding publications in international Science Citation Index (SCI) and Social Science Citation Index (SSCI) journals. We aim to address these research questions:

1. What is the current publication trend in knowledge hiding?
2. Which themes involving knowledge hiding have been studied by scholars?
3. What are the areas involving knowledge hiding that seem to require future research?

Previous authors have conducted reviews on knowledge hiding (e.g., Xiao and Cooke, 2019; Anand et al., 2020; de Garcia et al., 2020), which are valuable. However, the review of Xiao and Cooke (2019) is based on 52 articles and all of which are written in English or Chinese, and published over the period 1997–2017. Similarly, the review of Anand et al. (2020) is drawing on 52 studies. In their work, de Garcia et al. (2020) have reviewed a total of 57 articles that are published up to April 2018, and their study focuses on distinguishing knowledge hiding and knowledge hoarding from knowledge collection and donation perspectives. Our review differs from these previous works in terms of volume, timeframe, method and the analysis. First, we have combined bibliometric analysis, content analysis and descriptive analysis in this review, which allows for incorporating rich data with less interpretative or subjectivity biases. In contrast to previous reviews, we further overview the concepts and dimensions, antecedents, consequences, theoretical foundations, and influence mechanisms of knowledge hiding. In the meantime, we have included bigger volume of articles in this review. In so doing, we are able to complement the previous reviews, offering a more objective account of evolution of this research topic.

## Methodology

Our study has followed the systematic review process (Pickering and Byrne, 2014). Within this process, we employ the principles of Tranfield et al. (2003), which include (1) setting the scope, (2) conducting the search and data extraction, (3) selecting the studies and analyzing the data, and (4) extracting data and reporting the findings. To ensure the data validity and reliability, we limited our databases by searching the sample of English-written articles from the Web of Science over the period between 1995 and 2020. Further, the main reason for using SCI and SSCI databases is that web of science is “generally considered credible among the scientific community, and [are] commonly used by researchers from a wide range of fields (de Garcia et al., 2020, p. 4). Several reviews have used these databases (e.g., Bernatović et al., 2021; Vlačić et al., 2021).

**Figure 1:**
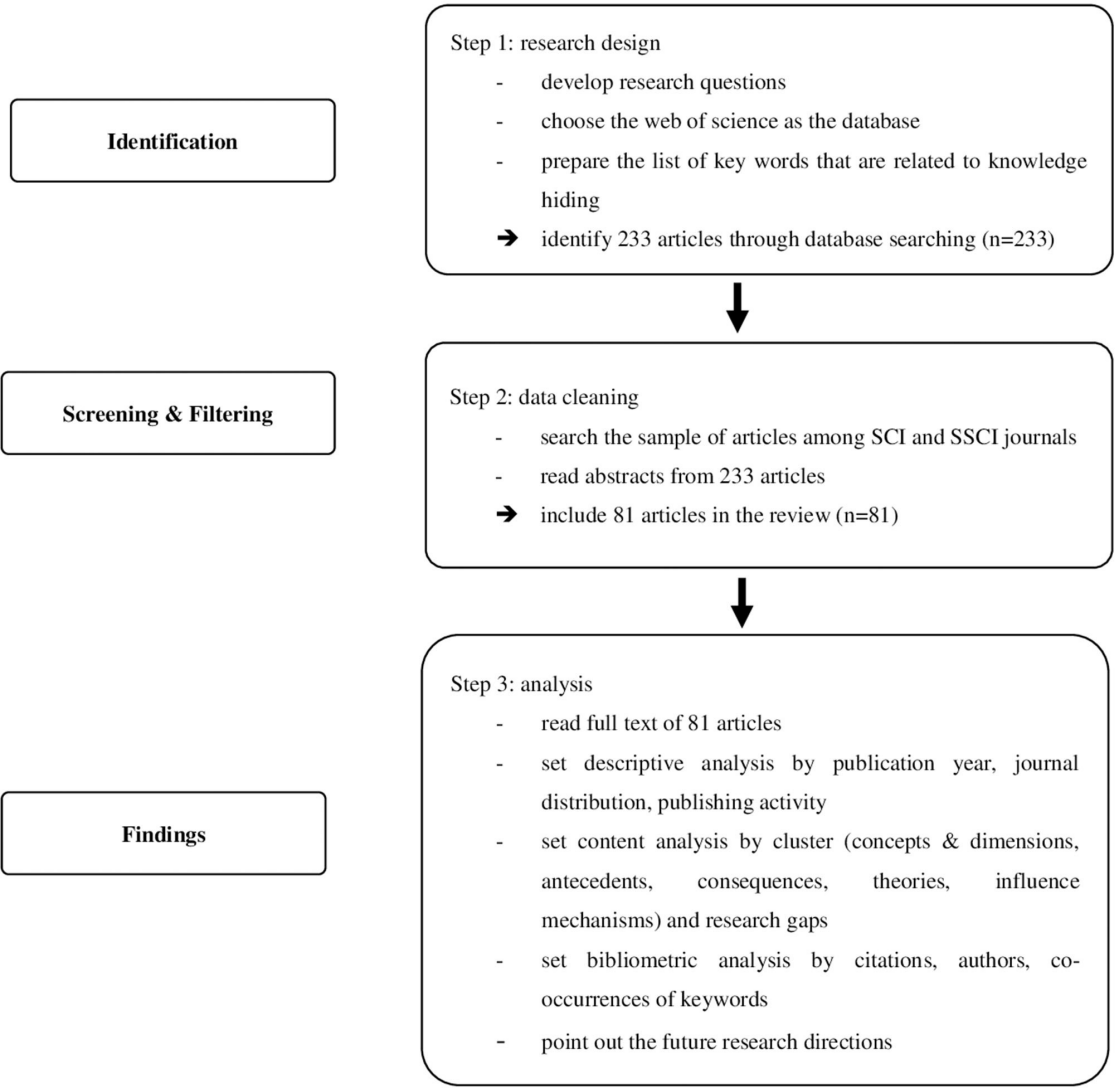
Flow diagram.

Retrieval conditions were “Title = knowledge hiding” or “Title = knowledge withholding,” and the time span was “All years (1950–2020).” The database was “Web of Science Core Collection” and the search basis was “Web of Science Category = Unrestricted Category.” In total, we obtained a sample of 233 articles. Subsequent analysis of these 233 articles' abstracts was conducted. In order to ensure data accuracy, we carefully selected studies that fit the definition given by Connelly et al. (2012) and excluded those that belonged to disciplines such as information management. This yielded 81 articles related to knowledge hiding. For these 81 articles, we undertook the reading of full texts, using Excel to record the key findings, theoretical lens, and methodologies. Building upon the content extraction, the authors classified the core clusters in five main themes according to their characteristics: concept and dimensions, antecedents, consequences, theoretical frameworks, and influence mechanism. Figure 1 shows the flow diagram of analysis.

## Analysis and Findings

### Publication by Year

**Figure 2:**
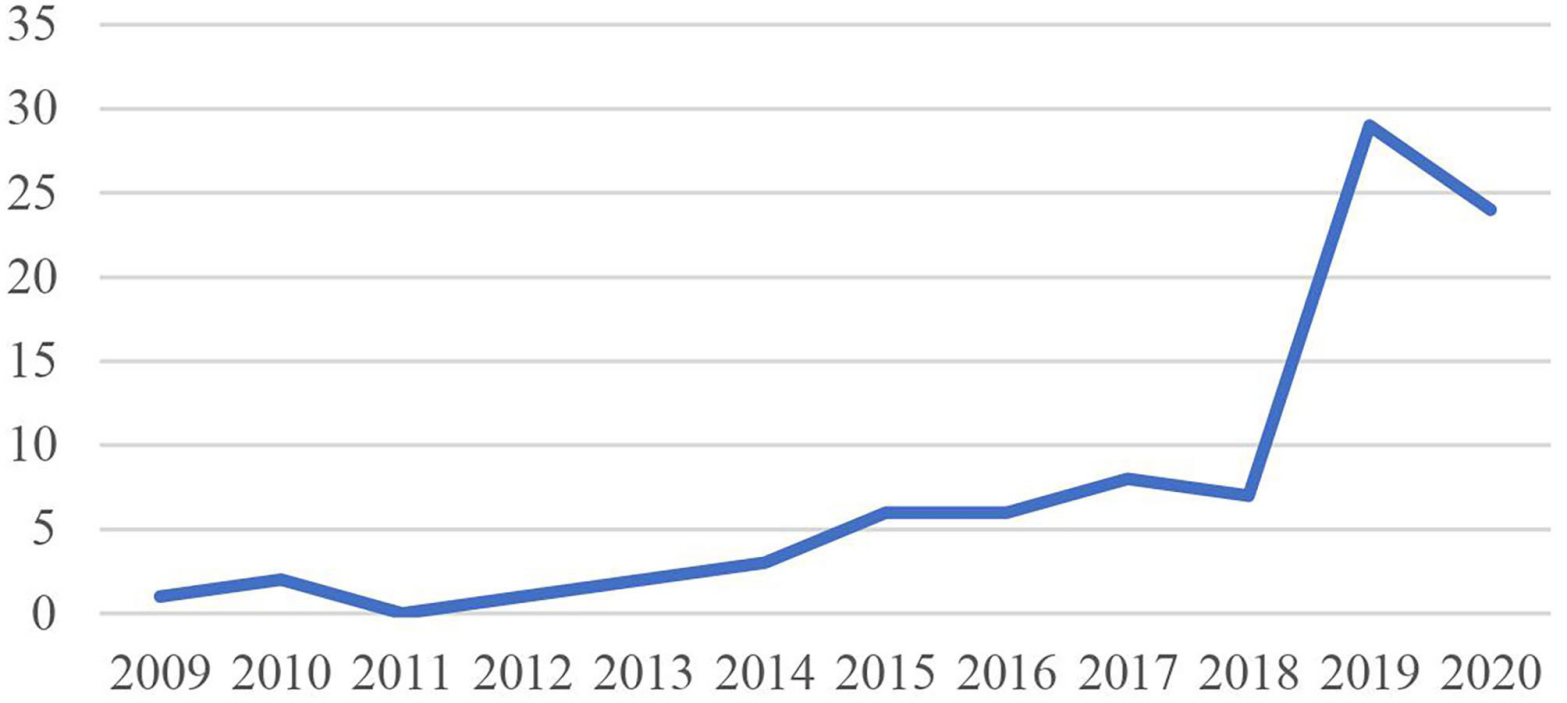
Annual distribution of articles on knowledge hiding.

The analysis of the number of publications per year on knowledge hiding in international journals (see Figure 2) shows that scholars started to systematically study knowledge hiding as an organizational behavior in the 2010s. A growing number of studies have addressed knowledge hiding but it dates back only to 2012, when knowledge hiding was first proposed as an independent concept in the work of Connelly et al. (2012). Knowledge hiding research has gone through two periods: the initial stage (from 2012 to 2018) and the fast development stage (from 2019 to 2020). During the initial stage, publications on knowledge hiding in mainstream international journals were rare, and there were only between one and five articles published per year. Since 2019, there has been a sharp increase in knowledge hiding publications; the number of publications has jumped to more than 30 articles per year (see Figure 2).

### Journal Distribution of Knowledge Hiding Research

From 2012 to 2020, research on knowledge hiding has been published in 43 SCI/SSCI journals (see Table 1), with 40 articles (49.38%) published in Journal Citation Reports (JCR) Q1 journals, 19 articles (23.46%) published in JCR Q2 journals, 8 articles (9.88%) published in JCR Q3 journals, and 11 articles (13.58%) published in JCR Q4 journals; 15 articles (18.52%) published in the Chartered Association of Business Schools (ABS3) journals, 10 articles (12.35%) published in ABS4 journals, one article (1.23%) published in Financial Times (FT50) journals; and one article (1.23%) published each in UT Dallas top 100 business school research rankings (UTD24) and ABS4^*^ journals. The top 10 journals that published most of the knowledge hiding articles are *Journal of Knowledge Management, Journal of Organizational Behavior, Management Decision, International Journal of Hospitality Management, European Journal of Work and Organizational Psychology, Knowledge Management Research and Practice, International Journal of Information Management, Asian Business and Management, Leadership and Organization Development Journal*, and *Journal of Managerial Psychology*. The majority of knowledge hiding research has been published in JCR Q1/Q2 journals, and a considerable proportion has been published in ABS3/4 journals.

**Table 1:** Top publishing journals on knowledge hiding.

**No**.	**Journal name**	**Journal information (Impact factor and ranking)**	**Number of publications**
1	Journal of Knowledge Management	IF: 4.745, JCR Q1	17
2	Journal of Organizational Behavior	IF: 5.026, JCR Q1, ABS 4	7
3	Management Decision	IF: 2.723, JCR Q2	4
4	International Journal of Hospitality Management	IF: 6.701, JCR Q1, ABS 3	3
5	European Journal of Work and Organizational Psychology	IF: 2.882, JCR Q2, ABS 3	3
6	Knowledge Management Research & Practice	IF: 1.583, JCR Q4	3
7	International Journal of Information Management	IF: 8.210, JCR Q1	2
8	Asian Business & Management	IF: 2.192, JCR Q3	2
9	Leadership & Organization Development Journal	IF: 1.977, JCR Q3	2
10	Journal of Managerial Psychology	IF: 1.380, JCR Q4, ABS 3	2
11	Academy of Management Journal	IF: 7.571, JCR Q1, UTD 24, ABS 4	1
12	International Journal of Project Management	IF: 6.620, JCR Q1	1
13	Computers in Human Behavior	IF: 5.003, JCR Q1, ABS 3	1
14	Journal of Business Research	IF: 4.874, JCR Q1, ABS 3	1
15	Decision Support Systems	IF: 4.721, JCR Q1, ABS 3	1
16	Industrial Marketing Management	IF: 4.695, JCR Q1, ABS 3	1
17	Journal of Business Ethics	IF: 4.141, JCR Q1, FT 50, ABS 3	1
18	Telematics and Informatics	IF: 4.139, JCR Q1	1
19	Academy of Management Learning & Education	IF: 4.058, JCR Q1, ABS 4	1
20	Human Resource Management Journal	IF: 3.816, JCR Q1, ABS 4	1
21	Higher Education	IF: 2.856, JCR Q1	1
22	International Entrepreneurship and Management Journal	IF: 3.472, JCR Q2	1
23	Applied Psychology: An International Review	IF: 2.808, JCR Q2, ABS 3	1
24	International Journal of Environmental Research and Public Health	IF: 2.849, JCR Q2	1
25	Journal of Occupational and Organizational Psychology	IF: 2.652, JCR Q2, ABS 4	1
26	Sustainability	IF: 2.576, JCR Q2	1
27	Project Management Journal	IF: 2.506, JCR Q2	1
28	Journal of the Association for Information Science and Technology	IF: 2.410, JCR Q2	1
29	Personality and Individual Differences	IF: 2.311, JCR Q2, ABS 3	1
30	Frontiers in Psychology	IF: 2.067, JCR Q2	1
31	Interactive Learning Environments	IF: 1.938, JCR Q2	1
32	International Journal of Conflict Management	IF: 1.806, JCR Q2	1
33	The Journal of Psychology	IF: 1.548, JCR Q2	1
34	Basic & Clinical Pharmacology & Toxicology	IF: 2.651, JCR Q3	1
35	The Service Industries Journal	IF: 2.381, JCR Q3	1
36	Asia Pacific Journal of Human Resources	IF: 1.894, JCR Q3	1
37	Baltic Journal of Management	IF: 1.719, JCR Q3	1
38	Journal of Nursing Management	IF: 2.243, JCR Q4	1
39	Negotiation and Conflict Management Research	IF: 1.027, JCR Q4	1
40	International Journal of Emerging Markets	IF: 1.022, JCR Q4	1
41	Journal of Organizational Change Management	IF: 0.967, JCR Q4	1
42	Sage Open	IF: 0.715, JCR Q4	1
43	Social Behavior and Personality	IF: 0.676, JCR Q4	1

### Publishing Activity by Authors, Authors' Institutions, and Locations

Knowledge hiding has attracted considerable attention from researchers and practitioners. As shown in Table 2, Matej Cerne published the most articles (eight) on knowledge hiding followed by Škerlavaj and Connelly, with seven and six articles respectively. The most active institutions in the research field of knowledge hiding were University of Ljubljana (eight publications), followed by BI Norwegian Business School, McMaster University and Tongji University, each with seven publications. Table 3 lists the locations of authors' institutions, with the top four being China, Pakistan, Canada and United Arab Emirates.

**Table 2:** Top publishing authors and institutions on knowledge hiding.

**Author**	**Total publication**	**Institution**	**Total publication**
Matej Cerne	8	University of Ljubljana	8
Miha Škerlavaj	7	BI Norwegian Business School	7
Catherine E. Connelly	6	McMaster University	7
Anders Dysvik	5	Tongji University	7
Jinlian Luo	5	United Arab Emirates University	6
Abdul Karim Khan	4	Shanghai University	5
Atif Saleem Butt	4	American University of Ras Al Khaimah	4
Hongdan Zhao	4	University of International Business and Economics	4
Muhammad Usman	4	University of Science and Technology of China	4
Usman Ghani	4	Zhejiang University	4
Ghulam Ali Arain	3	Brock University	3
Qing Xia	3	COMSATS University Islamabad	3
Sadia Jahanzeb	3	International Islamic University	3
Tasneem Fatima	3	Memorial University of Newfoundland	3
Xuesong Zhai	3	Tamkang University	3
		Wuhan University	3

**Table 3:** Publishing activity by authors' institution location.

**Institution location**	**Total publication**
China	40
Pakistan	16
Canada	11
United Arab Emirates	11
Slovenia	8
United States	7
Norway	7
Taiwan	6
Australia	5
Singapore	4
United Kingdom	3
Croatia	2
Turkey	2
India	2
Finland	2
Germany	2
Saudi Arabia	2
Italy	2
France	2
Iraq	2
Indonesia	1
Spain	1
Cyprus	1
Portugal	1
Austria	1
Switzerland	1
Brazil	1

### Publishing Activity by Data Sources

Our analysis shows that previous data on knowledge hiding have tended to be collected in one single location, such as China, Pakistan, United Arab Emirates, Saudi Arabia, United States, and so on (see Table 4). Eight publications used data that were collected from multi-countries and regions (e.g., North America, Germany and Austria, Europe, Slovenia, Croatia, Serbia, Bosnia and Herzegovina, Montenegro and Macedonia). The top three locations from which researchers have collected knowledge hiding data were China (29 publications), Pakistan (13 publications) and United Arab Emirates (5 publications).

**Table 4:** Locations from which researchers have collected knowledge hiding data.

**Knowledge hiding data collected in …**	**Total Publication**
China	29
Pakistan	13
^*^Multi-countries and regions	8
United Arab Emirates	5
^*^Literature review	4
Saudi Arabia	3
United States	3
Finland	2
India	2
Slovenia	2
Myanmar	2
Taiwan	2
Turkey	2
Canada	1
Indonesia	1
Jordan	1
^*^One European Union member state	1
Total	81

* *Multi-countries and regions = data were collected in more than one country*.

* *Among the 81 articles, four publications concerned literature review, and one publication has used data collected from one European Union member state but the country name was not indicated in the article*.

### Highly Cited Publications

Citations can show the research focus of scholars and reveal their main theoretical lens. Highly cited articles are often regarded as important references in the field. Table 5 presents the top 15 highly cited publications on knowledge hiding.

**Table 5:** Top 15 articles on knowledge hiding by the number of citations.

**No**.	**Article**	**Year of publication**	**Authors**	**Number of citations (as of 2020)**	**ESI highly cited (Yes/No)**
1	Understanding counterproductive knowledge behavior: Antecedents and consequences of intra-organizational knowledge hiding	2016	Serenko and Bontis	81	Yes
2	Evasive knowledge hiding in academia: When competitive individuals are asked to collaborate	2019	Hernaus, Cerne, Connelly, Vokic and Š-kerlavaj	24	Yes
3	Territoriality, task performance, and workplace deviance: Empirical evidence on role of knowledge hiding	2019	Singh	27	Yes
4	Perceptions of organizational politics, knowledge hiding, and employee creativity: The moderating role of professional commitment	2019	Malik, Shahzad, Raziq, Khan, Yusaf and Khan	19	Yes
5	Knowledge hiding in organizations	2012	Connelly, Zweig, Webster and Trougakos	455	No
6	What goes around comes around: Knowledge hiding, perceived motivational climate, and creativity	2014	Cerne, Nerstad, Dysvik and Škerlavaj	95	No
7	Why and when do people hide knowledge?	2013	Peng	94	No
8	How perpetrators and targets construe knowledge hiding in organizations	2015	Connelly and Zweig	89	No
9	Workplace ostracism and knowledge hiding in service organizations	2016	Zhao, Xia, He, Sheard and Wan	54	No
10	Antecedents and intervention mechanisms: A multi-level study of R&D team's knowledge hiding behavior	2016	Huo, Cai, Luo, Men and Jia	48	No
11	Hiding behind a mask? Cultural intelligence, knowledge hiding, and individual and team creativity	2017	Bogilović, Cerne and Škerlavaj	42	No
12	The role of multilevel synergistic interplay among team mastery climate, knowledge hiding, and job characteristics in stimulating innovative work behavior	2017	Cerne, Hernaus, Dysvik and Škerlavaj	56	No
13	Tell me if you can: Time pressure, prosocial motivation, perspective taking, and knowledge hiding	2018	Škerlavaj, Connelly, Cerne and Dysvik	32	No
14	Knowledge hiding and team creativity: The contingent role of task interdependence	2018	Fong, Men, Luo and Jia	30	No
15	When and how abusive supervision leads to knowledge hiding behaviors–An Islamic work ethics perspective	2018	Khalid, Bashir, Khan and Abbas	28	No

Further, through a co-citation analysis, co-authorship analysis, keyword and co-occurrence analysis, and content analysis, we find that most research on knowledge hiding focuses on the concept and dimensions of the topic. For instance, as one of the highly cited publications, it is important to acknowledge that Connelly et al. (2012) take the lead in defining the concept of knowledge hiding and propose evasive hiding, playing dumb, and rationalized hiding as three dimensions of knowledge hiding. Based on the work of Connelly et al. (2012); Zhao et al. (2016) further examine the interpersonal antecedents of the three dimensions of knowledge hiding. Hernaus et al. (2019) distinguish the three dimensions of knowledge hiding and address how individual competitiveness may lead to knowledge hiding. Connelly and Zweig (2015) point out that the three dimensions of knowledge hiding are not equally and always harmful, where under certain circumstances, some knowledge hiding can be beneficial. Among the highly cited publications, scholars also focus on the antecedents of knowledge hiding, paying particular attention to workplace stressors, psychological ownership, and territoriality of knowledge. For example, Zhao et al. (2016); Škerlavaj et al. (2018), and Khalid et al. (2018) have examined the influence mechanisms of workplace stressors, such as workplace ostracism, abusive supervision, and interpersonal injustice, on knowledge hiding. Peng (2013); Huo et al. (2016), and Singh (2019) emphasize the predictive effect of psychological ownership and territoriality of knowledge on knowledge hiding. Serenko and Bontis (2016); Hernaus et al. (2019), and Malik et al. (2019) also investigate the antecedents of knowledge hiding with different focuses (e.g., intra-organizational knowledge hiding, the individual-level and job-related factors within academia, organizational politics). These studies represent the two most important research directions of knowledge hiding.

Following, among the highly cited publications, we find that individual and team creativity, interpersonal relationships, and retaliation show the key consequences of knowledge hiding. The main contributions in the field include the work of Cerne et al. (2014), who point out that “when employees hide knowledge, they trigger a reciprocal distrust loop in which coworkers are unwilling to share knowledge with them” (p. 172). In recent years, Connelly and Zweig (2015), and Serenko and Bontis (2016) also prove that knowledge hiding can lead to retaliation. Cerne et al. (2017) and Malik et al. (2019) examine the destructive effect of knowledge hiding on individual creativity. Bogilović et al. (2017) and Fong et al. (2018) analyze the impacts of individual-level knowledge hiding on team-level creativity. These studies represent the mainstream consequences of knowledge hiding.

Additionally, we identify that the research focus on knowledge hiding has moved from the individual level to a multilevel influence mechanism. For example, Huo et al. (2016); Cerne et al. (2017); Fong et al. (2018), and Hernaus et al. (2019) explore the moderating effect of team-level task interdependence on the relationship between individual-level variables and knowledge hiding. In addition, team-level cultural factors (e.g., mastery climate, workplace ethics) and organizational justice are variables that scholars have examined when exploring the multilevel influence mechanism of knowledge hiding (Huo et al., 2016; Cerne et al., 2017; Khalid et al., 2018).

### Major Research Clusters and Topics

**Figure 3:**
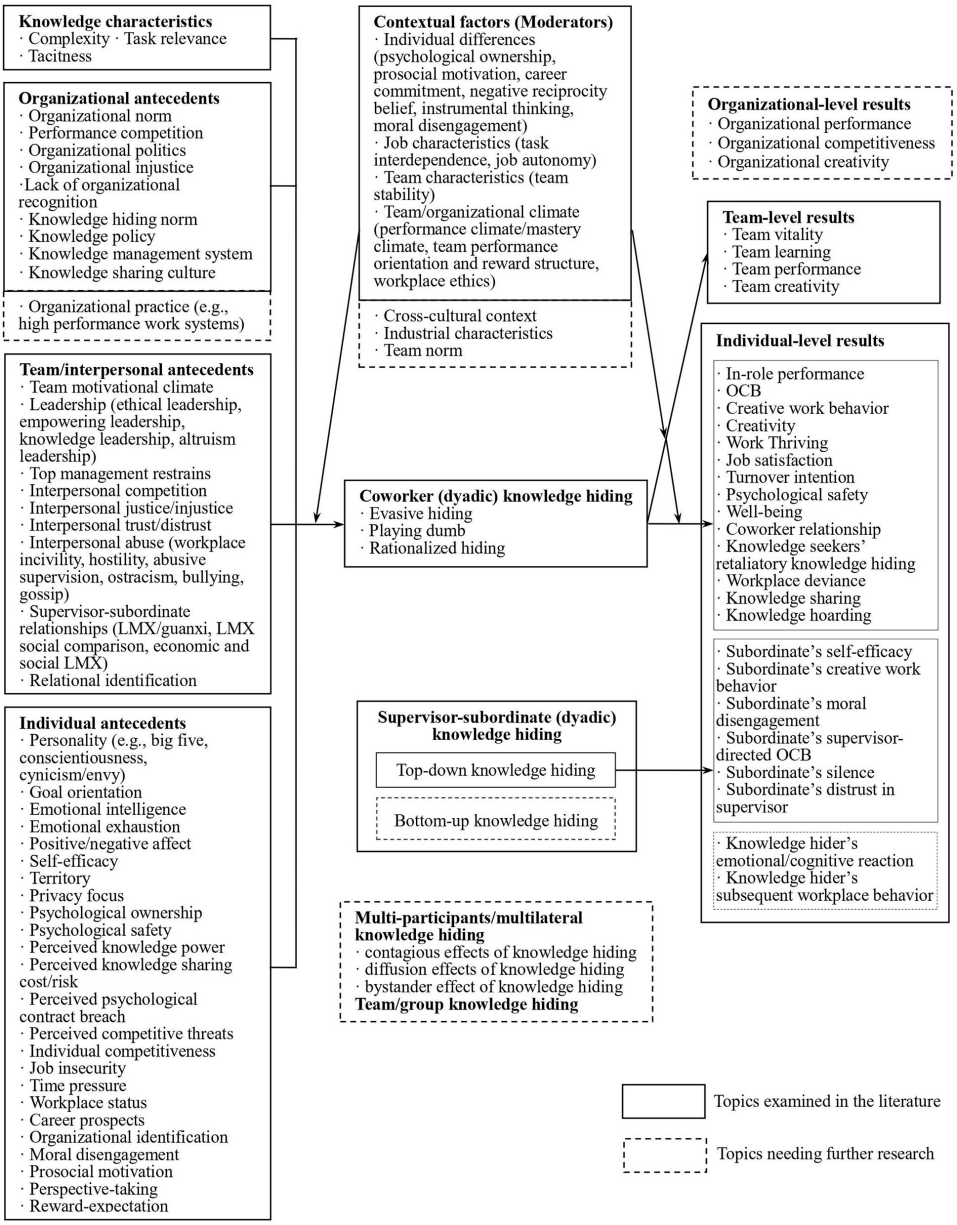
Research framework of knowledge hiding. Source: extended and developed from Connelly et al. (2012) and Xiao and Cooke (2019).

Using CiteSpace4.0 software, we conducted the descriptive analysis, bibliometric analysis, and content analysis of the 81 knowledge hiding articles that are published in the international journals from 2012 to 2020. In order to clearly demonstrate the current status of knowledge hiding research, we structure our findings into the following five clusters (see Figure 3).

#### Concept and Dimensions

The bibliometric analysis suggests that keywords related to the concept of knowledge hiding include knowledge sharing, knowledge withholding, and knowledge management process. Based on these keywords and the results of our content analysis, we extract “concept and dimensions” as the first cluster that reflects the research interests in knowledge hiding.

The concept of knowledge hiding was first defined as the act of deliberately not providing knowledge or providing knowledge that is not what the seeker needs when facing a colleague's request (Connelly et al., 2012). These were the first authors to discuss the linkages and differences between knowledge hiding and related concepts, such as knowledge sharing/non-sharing (Anand et al., 2020), knowledge withholding (Webster et al., 2008), knowledge hoarding (Xiao and Cooke, 2019; de Garcia et al., 2020), counterproductive/deviant behavior (Connelly and Zweig, 2015; Serenko and Bontis, 2016), workplace deception (Connelly et al., 2012), and incivility (Zhao et al., 2016). Later, scholars further proposed concepts such as knowledge sharing hostility (Stenius et al., 2016), disengagement from knowledge sharing (Zhao et al., 2016), knowledge contribution loafing (Fang, 2017), and knowledge manipulation (Bogilović et al., 2017). In recent years, scholars have tried to differentiate knowledge hiding from other related concepts (e.g., employee silence and knowledge protection) (Bari et al., 2020).

**Figure 4:**
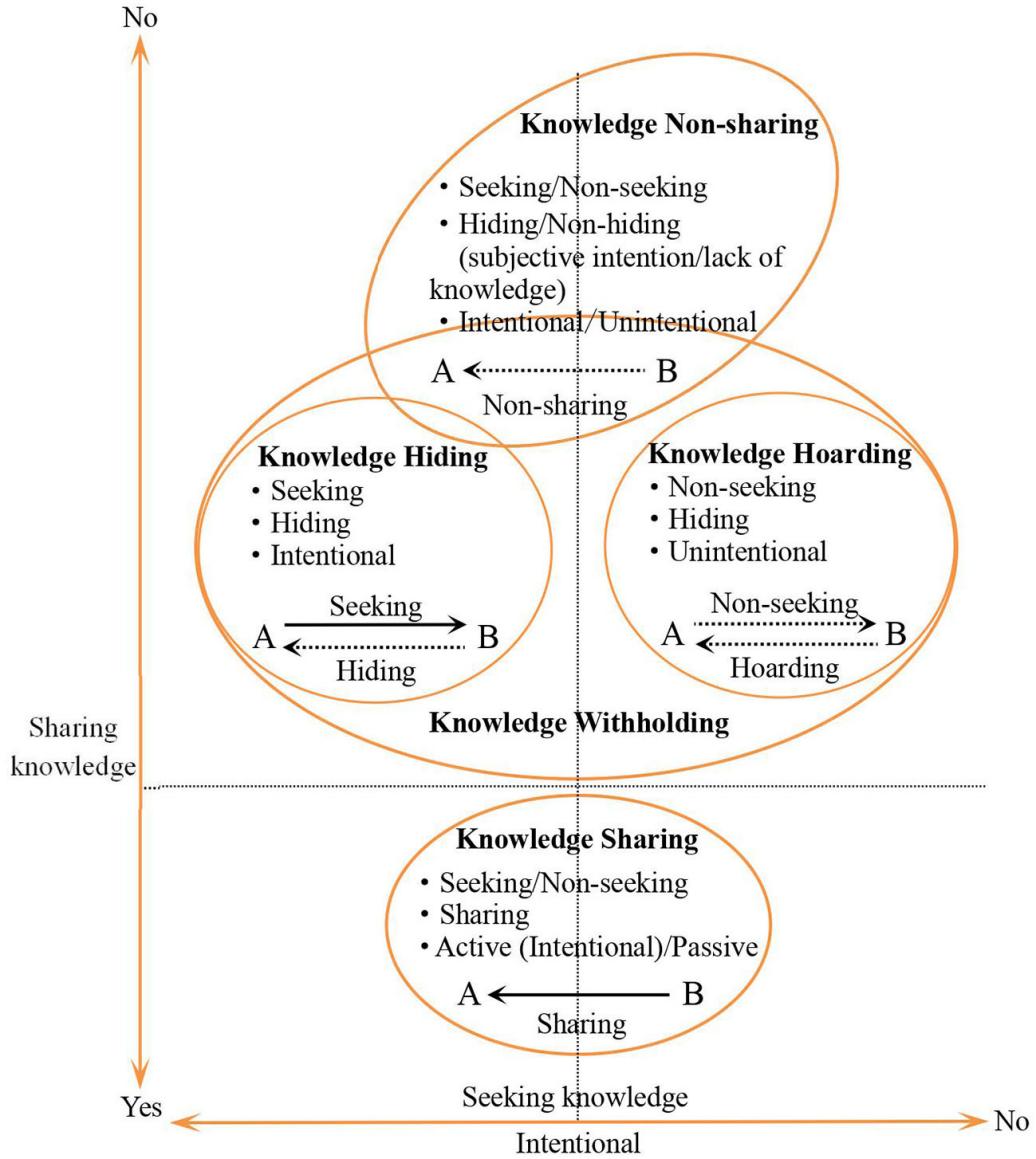
Comparison between knowledge hiding and related concepts. Source: extended and developed from Connelly et al. (2012) and de Garcia et al. (2020).

In order to distinguish these different concepts, we compare relevant concepts through questioning whether knowledge seeking exists, the degree of knowledge sharing, and the intentionality of the behavior (see Figure 4). In general, scholars have widely accepted the definition of knowledge hiding given by Connelly et al. (2012). The mainstream view believes that knowledge hiding is an important aspect of knowledge withholding, and it is not the opposite of knowledge sharing (Connelly et al., 2012; Serenko and Bontis, 2016; Zhao et al., 2016). Consequently, one cannot simply equate knowledge hiding with non-sharing or a lack of knowledge sharing. In addition to subjective intention, the reasons that individuals do not share knowledge with others can be related to a lack of relevant knowledge or the inability to share the knowledge. It is worth pointing out that there are different opinions in boundaries between knowledge hiding and concepts such as knowledge non-sharing, counterproductive knowledge behavior, and knowledge protection. Hence, there still exists some confusion and cross-use of related concepts in the knowledge hiding research. In addition, the existing literature has seldom defined knowledge hiding from the indigenous/cross-cultural perspective.

Connelly et al. (2012) have developed three dimensions of knowledge hiding and an employee self-evaluation scale with 12 items, with each dimension measuring four items. Among them, evasive hiding means that the hider provides invalid knowledge or pretends to agree to help, but lacks follow-up action. An example item is “I agreed to help him/her but never really intended to.” Playing dumb refers to pretending to be ignorant of the relevant knowledge or not understanding the knowledge seeker's question, with a sample item “I pretended I did not know what he/she was talking about.” Rationalized hiding means that the hider explains the reasons for not providing required knowledge, such as the necessity to keep it confidential or offering that knowledge sharing is not allowed by the superiors. An example item is “I explained that the information is confidential and available only to people on a particular project.” Most scholars believe that rationalized hiding is different in nature from evasive hiding and playing dumb, because rationalized hiding does not involve deception, but the evasive hiding and playing dumb do have a high degree of deception.

The scale of Connelly et al. (2012) has been proved to have high reliability and validity in a series of empirical studies. In general, scholars use this scale and its original items directly, making some contextual adaptation of expressions only according to the particular research needs. There are other knowledge hiding scales, such as Peng's (Peng, 2013) three-item counterproductive knowledge behavior scale and knowledge withholding behavior scales developed by Lin and Huang (2010); Tsay et al. (2014), and Serenko and Bontis (2016). Anand et al. (2020) have advocated that knowledge hiding is composed of unintentional hiding (driven by contingent situation), motivational hiding (driven by performance and competition), controlled hiding (driven by psychological ownership), victimized hiding (driven by hostility and abuse), and favored hiding (driven by identity and norms). Jha and Varkkey (2018) identify the four strategies adopted by supervisors to hide knowledge from subordinates, namely, playing innocent, misleading, rationalized hiding, and counter-questioning.

#### Antecedents

The antecedents of knowledge hiding include the Big Five personality traits, abusive supervision, negative workplace gossip, and career insecurity. Combined with the research framework of knowledge hiding (see Figure 3), the second cluster as antecedents is popular among scholars. Inspired by the work of Connelly et al. (2012) and Xiao and Cooke (2019), we review knowledge hiding antecedents from four aspects: knowledge characteristics, individual factors, team and interpersonal factors, and organizational factors.

Knowledge characteristic is one of the first antecedents popular among scholars. Due to the complex nature of knowledge, Connelly et al. (2012) point out that such complexity affects the willingness of individuals to provide help when facing colleagues' knowledge requests. Simply, it often requires more time and energy to generate complex knowledge that knowledge owners tend to keep the knowledge for themselves. Hernaus et al. (2019) argue that people are more likely to hide tacit knowledge rather than explicit knowledge. In addition, the task relevance and the value of knowledge have a positive relation with knowledge hiding (Connelly et al., 2012; Huo et al., 2016).

Individual factors mainly include personality traits and psychological factors such as emotion and cognition. In terms of personality traits, scholars focus mainly on the influence of the Big Five personality traits, in particular neuroticism. For example, Pan and Zhang (2018) reveal that employees with high conscientiousness and low neuroticism are less likely to hide knowledge, while people with high neuroticism are more likely to hide knowledge (Anaza and Nowlin, 2017). Pan et al. (2018) verify the effects of a “dark triad of personality” (Machiavellianism, narcissism, and psychopathy) on different dimensions of knowledge hiding. Fang (2017) and Aljawarneh and Atan (2018) examine the relationship between anxiety and knowledge hiding and the relationship between cynicism and knowledge hiding.

When it comes to the cognitive perception, prior research has focused mainly on the individual's self-efficacy, territoriality and psychological ownership, psychological safety, psychological contract breach, perceived pressure or job insecurity, perceived workplace status, and career prospects. Tsay et al. (2014); Jha and Varkkey (2018), and Hernaus et al. (2019) argue that individuals' confidence in their knowledge and perception of their competitiveness affect their willingness to share knowledge. Peng (2013); Huo et al. (2016); Kang (2016); Singh (2019); Khalid et al. (2020), and Zhai et al. (2020) believe individuals' perceived exclusivity of knowledge, knowledge power, and knowledge privacy are the primary factors that determine how much knowledge they are willing to share with colleagues. He et al. (2020); Lin et al. (2020), and Wu (2020) explore the formation mechanism of knowledge hiding from the perspectives of psychological safety and perceived threats. Pradhan et al. (2019); Ghani et al. (2020a), and Jahanzeb et al. (2020a) emphasize the negative impacts of employee psychological contract breaches on knowledge sharing in the organizations. Jha and Varkkey (2018); Škerlavaj et al. (2018), and Feng and Wang (2019) examine the impacts of workplace stressors, such as time pressure and job insecurity, on knowledge hiding.

Prior studies have also investigated knowledge hiding from employee and supervisor perspectives. In their work, Butt (2019) and Butt and Ahmad (2019) show that concerns about career prospects are important individual-level reasons for supervisors to hide knowledge from subordinates. Liu et al. (2020) find that perceived workplace status affects knowledge hiding through two opposing mechanisms: perception of knowledge sharing obligation and perception of being envied. The goal orientation has also attracted some scholars' attention in recent years when studying knowledge hiding behavior. Research by Zhu et al. (2019) shows that performance-driven goal orientation has a positive relationship with employees' knowledge hiding behaviors, which allows employees to achieve the competitive goal of surpassing colleagues. Nadeem et al. (2021) argue that shared goals are negatively related to knowledge hiding. Moh'd et al. (2021) analyze the relationship between achievement goal orientation (e.g., learning goals, performance display/performance-avoidance goal orientation) and knowledge hiding. Some scholars highlight that individual motivational factors (such as expected results/rewards and perceived knowledge sharing costs) affect knowledge hiding (Lin and Huang, 2010; Shen et al., 2019). Although emotion and cognition have been regarded as the two core elements that drive individual behavior (e.g., Lee and Allen, 2002), studies on how emotional/affective factors influence knowledge hiding are still underdeveloped. We believe only Zhao and Xia (2019) have studied the negative emotional state of nursing staff as the antecedent of their knowledge hiding behavior.

Team-level and interpersonal factors reflect leadership, interpersonal relationships, and their respective interactions. When considering leadership, scholars pay the most attention to abusive leadership, followed by ethical leadership. Khalid et al. (2018) point out that knowledge hiding is not necessarily an employee's intention to directly harm other organization members, but a negative reaction of employees to abusive supervision. Further, as indicated by displaced aggression theory, when employees encounter abusive leaders, they are more likely to retaliate by targeting innocent victims, namely, their colleagues but not the leaders. Based on the reactance theory, Feng and Wang (2019) point out that when employees experience frustration resulting from the abuse of their supervisors, they will take revenge in a direct or indirect way so that they can maintain a sense of freedom. However, because of their supervisors' supreme power and status in organizations, employees usually do not directly retaliate against supervisors so as not to cause stronger hostility and reciprocal retaliation. Ethical leadership can also influence employees' behavior intentionally or unintentionally through the role model effect. Abdullah et al. (2019); Anser et al. (2020), and Men et al. (2020) argue a significant but negative correlation between ethical leadership and subordinates' knowledge hiding behavior. Interestingly, the study by Xia et al. (2019) describes an inverted U–shaped curve relationship between knowledge leadership and employee knowledge hiding. Through a multilevel model, Lin et al. (2020) find that individual-focused empowering leadership can improve the supervisor-subordinate relationship and therefore inhibit knowledge hiding, whereas differentiated empowering leadership can cause group relational conflict and then lead to knowledge hiding. Based on social exchange theories, Abdillah et al. (2020) argue that altruistic leaders' humility, patience, understanding, sympathy, and compassion will be perceived by employees as uniquely socio-emotional resources, which can enhance the positive emotion of employees, improve the quality of the exchange between supervisors and subordinates (obtaining the trust and respect of the subordinates), and encourage employees be willing to make extra efforts for the organization and eliminate selfish behaviors that harm the interests of the organization, thus effectively preventing employee knowledge hiding behaviors.

From the perspective of interpersonal abuse, prior research shows that employees who encounter interpersonal unfair treatment are less willing to share their personal knowledge assets with others (Abubakar et al., 2019), whereas fair interpersonal interaction is significantly negatively correlated with the three dimensions of knowledge hiding (Ghani et al., 2020b). Among these, the factor of passive-aggressiveness in the workplace attracts more attention from scholars. Aljawarneh and Atan (2018) find that incivility in the workplace can drive employees to feel cynical and thus hide knowledge as a countermeasure. Zhao et al. (2016) and Riaz et al. (2019) point out that, as a typical workplace passive-aggressiveness, workplace ostracism would significantly increase employees' deceptive knowledge hiding (e.g., evasive hiding and playing dumb). Similarly, research by Yao et al. (2020a,b) shows that negative interpersonal experiences, such as workplace bullying and negative workplace gossip, accelerate the exhaustion of employee resources, such as emotions, time, energy, and organizational identity, leading them to hide knowledge. Anand et al. (2020) also find that hostility and abusive colleagues/supervisors drive employees to hide knowledge.

Concerning the impacts of interpersonal relationship on knowledge hiding, current research has focused on exploring the effects of supervisor-subordinate relationships. Scholars first divide supervisor-subordinate relationships into formal work-related relationships (contractual relationship, Leader-Member Exchange) and informal non-work-related relationships (Chinese personal *guanxi* relationships, Supervisor-Subordinate Guanxi) (He et al., 2020), or into economic LMX and social LMX (Babič et al., 2019), and then explore their impacts on employees' knowledge hiding behaviors. Previous research reveals that LMX negatively affects evasive hiding and playing dumb (Zhao et al., 2019). However, this reciprocal social exchange is more likely to reduce the level of knowledge hiding within the team, especially when the relationship between individuals and their supervisors has social LMX characteristics (Cerne et al., 2014). Furthermore, upward LMX social comparison leads to envy among team members, so it is a potential interpersonal antecedent of knowledge hiding among colleagues (Weng et al., 2020). It is worth noting that team prosocial motivation and social LMX (but not economic LMX) have an interaction effect on knowledge hiding (Babič et al., 2019). Lin and Huang (2010); Butt (2019); Butt and Ahmad (2019); Semerci (2019) examine the influences of interpersonal factors such as trust, reciprocity, relationship recognition, lack of interpersonal relationship, relationship conflict, and interpersonal competition. Interestingly, Lin and Huang (2010) point out that emotional bonds such as trust and reciprocity among team members can make individuals give up hiding too much knowledge to avoid retaliation from others. In addition, task conflicts and relationship conflicts have additive effects on knowledge hiding (Semerci, 2019).

At the organizational level, scholars have explored the roles of organizational culture, knowledge management policies and systems, organizational politics, organizational justice, organizational recognition, and a competitive performance environment on employees' conduct of knowledge hiding. First, the knowledge sharing culture has been proved to be closely related to the extent to which the knowledge hiding behavior can be accepted and adopted by the members of the organization (Connelly et al., 2012). For example, Anaza and Nowlin (2017) point out that the lack of incentives for knowledge sharing and the lack of supervisor feedback on subordinates' knowledge sharing will lead employees to hide knowledge. Jha and Varkkey (2018) highlight that a lack of organizational recognition of knowledge sharing and workload increase due to knowledge sharing increase employee knowledge hiding.

Social norms, organization policies, and management systems have also been found to have a profound impact on employees' tendency to hide knowledge. For instance, Butt and Ahmad (2019) argue that knowledge hiding is deeply embedded in many local companies and is regarded as a common code of conduct in the United Arab Emirates. Serenko and Bontis (2016) find that organizational knowledge management systems and policies have a significant direct impact on employee knowledge hiding, whereas injustice prompts employees to spontaneously engage in knowledge hiding behavior. Malik et al. (2019) propose that perceived organizational politics positively predict knowledge hiding. Abubakar et al. (2019) find that distributional, procedural, and interactional injustice increase the level of knowledge hiding among employees. Research by Jahanzeb et al. (2020b) confirms that employees who encounter organizational unfairness consider knowledge hiding as a means to rationalize the cognitive separation between oneself and the organization in order to maintain one's dignity. Finally, some scholars have examined the impact of a competitive working environment. For example, Anaza and Nowlin (2017) explain how internal competition can lead to knowledge hiding. Similar findings can be found in the work of Anand et al. (2020), who argue that organizational internal performance and competitive factors drive employees to hide knowledge.

#### Consequences

Based on the highly cited publications and the keyword analysis, we find that *consequences, performance, behavior*, and *employee/team creativity* are some keywords that reflect the outcome of knowledge hiding. Therefore, we use the term *consequences* to summarize the third cluster concerning the knowledge hiding research.

Current research focuses mainly on the individual- and team-level consequences of knowledge hiding. A small number of studies examine the individual-level consequences of knowledge hiding between supervisors and subordinates. In terms of individual-level results, the existing research has examined the effects of knowledge hiding on individual job performance, psychological status and attitude, workplace behavior, and supervisor-subordinate/coworker relationships. For instance, most studies have found that knowledge hiding among colleagues and between supervisors and subordinates can reduce task performance, organizational citizenship behavior (OCB), and creativity (Connelly et al., 2012; Cerne et al., 2014; Arain et al., 2019, 2020a,b; Jahanzeb et al., 2019; Malik et al., 2019; Singh, 2019; Zhu et al., 2019).

However, there are some mixing findings. For example, Wang et al. (2019) argue that perceived colleague knowledge hiding does not reduce the performance of salespersons. Instead, it encourages them to work harder to improve their sales performance. Burmeister et al. (2019) find that knowledge hiding (playing dumb, in contrast to evasive hiding and rationalized hiding) has opposite effects on OCB, and knowledge hiders experience different emotions. Khoreva and Wechtler (2020) point out that evasive hiding is negatively related to in-role performance, and playing dumb is positively related to it. In addition, both evasive hiding and rationalized hiding will hinder innovation performance. Regarding psychological status and attitudes, research suggests that knowledge hiding increases employees' moral disengagement (Arain et al., 2020a) and decreases their psychological safety, well-being, job satisfaction, and sense of thriving (Jiang et al., 2019; Offergelt et al., 2019; Khoreva and Wechtler, 2020). Furthermore, knowledge hiding can trigger knowledge seekers' deviant behaviors, turnover intention, upward silence, and non-engagement in knowledge sharing (Connelly and Zweig, 2015; Offergelt et al., 2019; Singh, 2019; Arain et al., 2020a).

Concerning interpersonal relationships, studies reveal that knowledge hiding among colleagues or between supervisors and subordinates can damage workplace relationships, which can even lead to a trust crisis (Connelly et al., 2012; Cerne et al., 2014; Arain et al., 2020b). In particular, Connelly et al. (2012), Cerne et al. (2014), and Connelly and Zweig (2015) highlight that knowledge hiding can result in a vicious circle of rejecting knowledge sharing. Studies also find that knowledge hiding has significant negative effects on team performance (Zhang and Min, 2019), team creativity (Fong et al., 2018; Bari et al., 2019), team viability (Wang et al., 2019), team learning, and absorptive capability (Fong et al., 2018; Zhang and Min, 2019).

In summary, scholars have made advancements on the impacts of knowledge hiding on the individual level, but research on its impacts on team and organizational levels is still at a nascent stage. Few scholars have recently analyzed the “boomerang effect” or “negative reinforcement cycle” of knowledge hiding—the impact of knowledge hiding on the hiders' psychological status, job performance, and creativity (e.g., Cerne et al., 2014; Jiang et al., 2019)—and its double-edged sword effect (Wang et al., 2019), which has opened up a new avenue for research.

#### Theoretical Perspectives

The fourth cluster concentrates on theories that are popular among scholars that they use to conduct knowledge hiding research. The theories applied in the field of knowledge hiding are mainly from two domains—managerial theory and psychological theory—and include theories such as “exchange” (represented by social exchange theory), “resources” [represented by Conservation of Resources (COR) Theory], “learning” (represented by social learning theory), “cognition” (represented by social cognitive theory), “ownership” (represented by psychological ownership theory), “goal orientation” (represented by achievement goal theory), “personality traits,” “job characteristics,” social identity theory, displaced aggression theory, and justice theory (see Table 6). Although scholars have introduced other theories to study knowledge hiding, the effectiveness of this theoretical development needs to be enhanced. For example, how to theorize individual emotions has not yet been made systematic and thus needs to be further explored in future research. Furthermore, we find that theories that are mostly used to examine the motivation/antecedents of knowledge hiding or the direct/indirect (mediating) influence of antecedent variables on knowledge hiding are less used to illustrate the consequences of knowledge hiding and the boundary conditions.

**Table 6:** Theoretical perspectives used in knowledge hiding research.

**Theories**	**Articles**	**Theory application in research**				
		**Antecedents/Motivations of knowledge hiding**	**Consequences of knowledge hiding**	**Direct effect**	**Mediating effect**	**Moderating effect**
Social exchange theory	Lin and Huang (2010), Connelly et al. (2012), Cerne et al. (2014), Tsay et al. (2014), Wang et al. (2014), Serenko and Bontis (2016); Bogilović et al. (2017), Fong et al. (2018), Khalid et al. (2018), Abdullah et al. (2019), Abubakar et al. (2019), Babič et al. (2019), Bari et al. (2019), Butt and Ahmad (2019), Jahanzeb et al. (2019), Pradhan et al. (2019), Semerci (2019), Singh (2019), Wang et al. (2019), Abdillah et al. (2020), Anand et al. (2020), He et al. (2020), Khalid et al. (2020), Lin et al. (2020), Ghani et al. (2020a), Arain et al. (2020b), and Nadeem et al. (2021)	√	√	√	√	√
Norm of reciprocity	Zhao et al. (2016), Singh (2019), and Arain et al. (2020b)	√	√	√		√
Conservation of resources theory	Aljawarneh and Atan (2018), Škerlavaj et al. (2018), Feng and Wang (2019), Riaz et al. (2019), Semerci (2019), Anand et al. (2020), Anser et al. (2020), Jahanzeb et al. (2020a), and Yao et al. (2020a,b)	√	√	√	√	√
Job demands-resources model	Malik et al. (2019)	√	√	√		
Social learning theory	Abdullah et al. (2019), Arain et al. (2019), Butt and Ahmad (2019), Offergelt et al. (2019), Zhao and Xia (2019), Anand et al. (2020), Lin et al. (2020), Ghani et al. (2020b)	√	√	√	√	√
Organizational learning theory	Zhang and Min (2019)		√		√	
Social cognitive theory	Lin and Huang (2010), Tsay et al. (2014), He et al. (2020), Arain et al. (2020a), Ghani et al. (2020b)	√	√	√	√	
Cognitive evaluation theory	Xia et al. (2019)	√		√		
Self-perception theory	Jiang et al. (2019)		√		√	
Moral disengagement theory	Zhao et al. (2016)	√				√
Psychological ownership theory	Peng (2013), Huo et al. (2016), Aljawarneh and Atan (2018), Abubakar et al. (2019), Singh (2019), and Anand et al. (2020)	√		√	√	√
Territoriality theory	Peng (2013) and Huo et al. (2016)	√			√	
Achievement goal theory	Cerne et al. (2014), Cerne et al. (2017), and Moh'd et al. (2021)	√	√	√		√
Goal orientation theory	Zhu et al. (2019)	√		√		
Personality traits theory	Wang et al. (2014)	√		√		
Trait activation theory	Pan and Zhang (2018)	√		√		
Cognitive-affective system theory of personality	Yao et al. (2020a,b)	√			√	
Job characteristic theory	Cerne et al. (2017) and Zhang and Min (2019)		√			√
Job design theory	Moh'd et al. (2021)	√		√		
Affect-as-information theory	Zhao and Xia (2019)	√			√	
Moral emotion theory	Burmeister et al. (2019)		√		√	
Displaced aggression theory	Khalid et al. (2018), Jahanzeb et al. (2019), Pradhan et al. (2019), and Ghani et al. (2020a)	√		√	√	
Social identity theory	Wang et al. (2014), Butt and Ahmad (2019), Zhao et al. (2019), and Jahanzeb et al. (2020b)	√		√	√	
Social comparison theory	Lin et al. (2020) and Weng et al. (2020)	√		√	√	
Social categorization theory	Bogilović et al. (2017) and Anand et al. (2020)		√			√
Social influence theory	Anand et al. (2020)	√				
Justice theory	Pradhan et al. (2019) and Jahanzeb et al. (2020b)	√		√	√	
Self-determination theory	Gagné et al. (2019) and Wang et al. (2019)	√	√	√	√	
Regulatory focus theory	Cerne et al. (2014) and Fang (2017)	√	√		√	√
Theory of planned behavior	Butt and Ahmad (2019)	√		√		
Theory of reasoned action	Wu (2020)	√		√	√	
Attribution theory	Khalid et al. (2020)	√				√
Protection motivation theory	Wu (2020)	√		√	√	
Psychological contract theory	Pan et al. (2018)	√			√	
Reactance theory	Feng and Wang (2019)	√		√		
Absorptive capacity theory	Fong et al. (2018)		√		√	
Cooperation-competition theory	Hernaus et al. (2019)	√		√		√
Status attainment theory	Liu et al. (2020)	√		√	√	
Agency theory	Khoreva and Wechtler (2020)		√	√		
Stimulus-organism-response (SOR) paradigm	Zhai et al. (2020)	√	√	√		
Interdependence theory	Connelly et al. (2012)	√		√		
Theory of basic values	Semerci (2019)	√				√
Broaden-and-build theory	Connelly and Zweig (2015) and Abdillah et al. (2020)	√	√	√		

#### Influence Mechanisms

There are findings on the mediating roles of antecedent variables that affect knowledge hiding. Emotional and cognitive factors (e.g., leadership, workplace stressors, interpersonal relationships, personality traits, and psychological ownership) can induce knowledge hiding. In terms of leadership, Abdullah et al. (2019) point out that ethical leadership inhibits employees' knowledge hiding by enhancing their relational social capital. Anser et al. (2020) find that the ethical behavior of ethical leaders can enhance the perception of “meaningful work” for service industries employees, thereby reducing the possibility of engaging in knowledge hiding behaviors. Khalid et al. (2018) find that perception of interpersonal justice mediates the relationship between abusive supervision and knowledge hiding. Feng and Wang (2019) believe that abusive supervision indirectly affects knowledge hiding through job insecurity. Pradhan et al. (2019) show that psychological contract breaching and the attacks toward supervisors play a partial mediating role in the process in which abusive supervision affects knowledge hiding. Ghani et al. (2020a) further point out that abusive supervision can easily lead to psychological contract breach, thus leading employees to attack their colleagues and deliberately hide knowledge from them. In addition, Lin et al. (2020) find that individual-focused empowering leadership enhances the psychological safety of subordinates, thereby reducing their knowledge hiding, whereas differentiated empowering leadership causes group relational conflicts, thereby increasing subordinate knowledge hiding. Abdillah et al. (2020) study the dual mediating mechanisms of altruistic leadership, which inhibits and prevents employees from knowledge hiding, pointing out that the positive emotions induced by altruistic leadership and LMX have important effects.

Regarding workplace stressors and interpersonal relationships, Aljawarneh and Atan (2018) find that cynicism mediates the relationship between tolerance of workplace incivility and knowledge hiding. Riaz et al. (2019) find that workplace ostracism has a significant impact on evasive hiding and playing dumb, and that work strain plays a mediating role. Yao et al. (2020a,b) have shown that relational identification and interpersonal trust play a chain-mediating role in the relationship between negative workplace gossip and knowledge hiding. At the same time, emotional exhaustion and organizational identification play a chain-mediating role in the relationship between workplace bullying and knowledge hiding. Jahanzeb et al. (2020b) believe that the experience of injustice causes employees to be psychologically separated from the organization and thus employees will show more knowledge hiding behaviors. Zhao et al. (2019) demonstrate that organizational identification mediates the negative impact of LMX on evasive hiding and playing dumb. Weng et al. (2020) point out that employees' upward LMX social comparison with their colleagues leads to envy of and knowledge hiding toward their colleagues. He et al. (2020) discover that psychological safety fully mediates the influence of LMX on knowledge hiding and partially mediates the influence of supervisor-subordinate *guanxi* on knowledge hiding.

Another aspect is shown through personality traits. Wang et al. (2014) find that perceived social identity mediates the relationship between the Big Five personality traits and knowledge hiding. Pan et al. (2018) examine the positive relationship between the “dark triad of personality” (Machiavellianism, narcissism, and psychopathy) and knowledge hiding, as well as the mediating effect of transactional psychological contracts on this relationship. Zhao and Xia (2019) point out that the negative affect states of nurses staff can “activate” their moral disengagement mechanism, allowing them to redefine their knowledge hiding behaviors as reasonable and acceptable, and thus exacerbating their knowledge hiding tendency. The final aspect is psychological ownership. Research by Peng (2013) and Huo et al. (2016) show that employees' psychological ownership of knowledge enhances their territorial awareness, which in turn causes them to hide knowledge from colleagues. Liu et al. (2020) confirm that the influence of workplace status on employee knowledge hiding is carried out through two opposite mechanisms: perceived knowledge sharing responsibility and envy. The former negatively mediates the relationship between the two, and the latter positively mediates it.

Some scholars have also studied the mediating effect of knowledge hiding. For instance, scholars examine the process through which knowledge hiding impairs individual or team creativity and innovation performance. Cerne et al. (2014) find that the knowledge hiding makes hiders reduce their own creativity, and colleague distrust plays a mediating role. Arain et al. (2019) show that supervisor knowledge hiding can reduce subordinates' self-efficacy and thus reduce their innovation. Khoreva and Wechtler (2020) point out that playing dumb and rationalized hiding can indirectly influence employee innovation performance through the mediating effect of well-being. Fong et al. (2018) confirm that a decrease in absorptive capacity is the key mediator in the relationship between knowledge hiding and team creativity. Zhang and Min (2019) state that team learning partially mediates the relationship between knowledge hiding and project team performance.

Moreover, researchers have studied the process through which knowledge hiding affects employees' subsequent interpersonal behaviors. For instance, Burmeister et al. (2019) find that guilt and shame play opposite mediating roles in the relationship between individual knowledge hiding and its subsequent interpersonal-oriented OCB. Arain et al. (2020b) point out that supervisor knowledge hiding negatively influences subordinates' OCB toward their supervisors, and subordinate distrust in their supervisors plays a mediating role. Supervisor knowledge hiding can also activate employee moral disengagement, prompting them to reduce OCB toward their supervisors and increase silence behaviors (Arain et al., 2020a). Jiang et al. (2019) suggest that knowledge hiding makes the hiders feel the insecurity of self-expression and interpersonal risk, thereby reducing their psychological safety and endangering their ability to thrive at work. Despite these advancements, it is necessary to develop a robust framework that integrates multipath models based on different innovative theoretical perspectives.

Regarding the moderating role of contextual factors on knowledge hiding, the existing research mainly explores the contingency influence of individual differences, job characteristics, team characteristics, and team/organizational climate. In terms of individual differences, some scholars find that organizational psychological ownership can effectively reduce the knowledge hiding resulting from territoriality (Peng, 2013). Furthermore, psychological ownership significantly moderates the inverted U-shaped relationship between knowledge leadership and knowledge hiding. This curved relationship is more obvious among employees with high psychological ownership (Xia et al., 2019). High psychological ownership can also minimize the impact of abusive supervision on knowledge hiding (Ghani et al., 2020a). Other scholars explore the boundary effect of positive traits, such as individualism/collectivist values (Semerci, 2019), positive affectivity (Jahanzeb et al., 2020a), benevolence or tolerance (Jahanzeb et al., 2020b), prosocial motivations (Škerlavaj et al., 2018), harmonious work enthusiasm (Anser et al., 2020), professional commitment (Malik et al., 2019), trust-related affect/cognition (Nadeem et al., 2021), social skills (Wang et al., 2019), and cultural intelligence (Bogilović et al., 2017). In addition to these studies, scholars examine the impacts of negative traits on knowledge hiding, such as negative reciprocity (Zhao et al., 2016; Jahanzeb et al., 2019), instrumental thinking (Abdullah et al., 2019), hostile attribution bias (Khalid et al., 2020), moral disengagement (Zhao et al., 2016), and cynicism (Jiang et al., 2019).

In relation to job characteristics, task interdependence has attracted a lot of attention. Huo et al. (2016) point out that task interdependence can reduce the territorial awareness and knowledge hiding caused by psychological ownership. Hernaus et al. (2019) find that task interdependence can help reduce the probability of employees' evasive knowledge hiding due to maintaining their competitiveness. Fong et al. (2018) show that task interdependence moderates the relationship between knowledge hiding and team absorptive capacity. Weng et al. (2020) suggest that the interdependence of cooperative and competitive goals has opposite moderating effects on the relationship between upward LMX social comparison and knowledge hiding. In addition, Pan and Zhang (2018) also analyze the influence of work autonomy on the intensity of the relationship between neuroticism and knowledge hiding.

Regarding the team/organizational climate, research shows that in an environment that values information exchange and cooperation, the negative influence of knowledge hiding will be greatly weakened. Accordingly, Cerne et al. (2014) study the boundary effect of the team achievement-motivation climate (e.g., performance climate and mastery climate) on the relationship between knowledge hiding and the decrease in the hider's creativity. They discover that the negative effect of knowledge hiding on the hider's creativity is reduced in a mastery climate. Furthermore, Cerne et al. (2017) find the moderating effects of mastery climate, task interdependence, and autonomy on the relationship between knowledge hiding and innovative work behavior. Bari et al. (2019) obtain similar findings which point out that a perceived mastery climate reduces the negative impact of evasive hiding and playing dumb on team creativity. Feng and Wang (2019) find that the interaction between abusive supervision and a mastery climate is negatively related to knowledge hiding, and the interaction between abusive supervision and a performance climate is positively related to knowledge hiding. On the one hand, when the organization pays more attention to individual performance feedback, performance-prove goal orientation can positively predict knowledge hiding. On the other hand, when the organization pays more attention to group performance feedback, performance-prove goal orientation is negatively correlated with knowledge hiding (Zhu et al., 2019). Compared to individual rewards, team-based rewards are more likely to reduce the distrust caused by knowledge hiding, promoting the team to work hard to achieve a common goal, forming a relatively stable team structure, and improving team viability (Wang et al., 2019). Yao et al. (2020a,b) reveal the buffering effect of a forgiveness climate on the relationship between negative workplace gossip/workplace bullying and knowledge hiding. Khalid et al. (2018) clarify the role of Islamic work ethics in moderating the relationship between abusive supervision and knowledge hiding. Among these findings, the existing research on the moderating effects still focuses more on the first stage of the antecedents–knowledge hiding–consequences linkage, but there is a lack of systematic development of the moderation mechanism in the second stage.

## Future Research Directions

Based on a descriptive analysis, bibliometric analysis, and content analysis, we find that research on knowledge hiding focuses mainly on five clusters. Despite the ongoing progress, several research gaps are worth further addressing.

(1) Comprehensive studies on the concept and dimensions of knowledge hiding are needed to provide a robust conceptual framework. Although the definition and three-dimensional view of knowledge hiding by Connelly et al. (2012) are widely adopted by many scholars, more research is needed to carry out in-depth comparative analysis to clarify the connections and differences between knowledge hiding and similar concepts (e.g., knowledge non-sharing, knowledge sharing hostility, knowledge contribution loafing, counterproductive knowledge behavior, knowledge hoarding, knowledge protection, employee silence, etc.). Further, more studies should continue exploring the dimensions of knowledge hiding. There is a lack of focus on knowledge hiders' psychological motivation and respective knowledge hiding strategies. For example, research on proactive, reactive, and passive knowledge hiding could enrich the field research. In addition, more studies should further explore the unique reasons and consequences of a rationalized hiding behavior. There is a need to verify the ethical aspect of rationalized hiding, when knowledge hiding is used to protect confidential information or the interests of third parties (Zhao et al., 2019).
(2) Future studies need to further explore the consequences of knowledge hiding. Based on a systematic review (see Figure 3), we find that previous studies have focused mainly on the antecedents of knowledge hiding. Although some studies have addressed the impacts of knowledge characteristics, individual factors, team-level and interpersonal factors, and organizational-level factors on knowledge hiding, more work is needed to provide comprehensive studies on the generating mechanisms and the respective coping strategies of knowledge hiding. Prior studies have shown that knowledge hiding has impacts on individual-level outcomes (e.g., individual creativity, in-role and extra-role performance, and coworker relationships) and team-level outcomes (e.g., team creativity). However, there is a lack of research on organizational-level outcomes. Moreover, prior studies focus mainly on the impacts of knowledge hiding on the knowledge seekers and the whole team, but seldom has the research discussed the potential effects of knowledge hiding on the knowledge hiders themselves. Therefore, future research should devote more attention to the negative effects of knowledge hiding on the knowledge hiders, the team, and the organization, and also explore the consequences of different dimensions of knowledge hiding. For example, more studies could address the research gap as to whether knowledge hiding may stimulate self-reflection and prompt moral and psychological compensation for the knowledge hiders. To enrich the multilevel mediating and moderating variables, future studies could explore the boundary conditions of knowledge hiding and their respective knowledge management strategies. In short, it is necessary to increase research on the consequences of knowledge hiding to enrich the antecedents–knowledge hiding–consequences research path.
(3) More studies on multilateral, cross-level, and collective knowledge hiding are needed, and it is appropriate to introduce new paradigms for knowledge hiding research. Existing research on knowledge hiding highlights mainly two parties: the hider (A) and the seeker (B) (i.e., B seeks knowledge from A, while A hides knowledge from B). Most studies address knowledge hiding among colleagues at the horizontal level. In recent years, some scholars have started to show interest in knowledge hiding at the vertical level, that is, the top-down knowledge hiding of superiors from subordinates. However, the research on the antecedents and the generating mechanisms of knowledge hiding at the vertical level is still in the stage of exploration. There is a lack of research on bottom-up knowledge hiding (of the subordinates from their superiors). Therefore, it is necessary to study knowledge hiding adopted by people from different hierarchies (e.g., bottom, mid, and high levels) in the organizations, comparing the differences between top-down and bottom-up knowledge hiding, so as to identify regular patterns of cross-level knowledge flow within the organizations. Future research could also examine whether the knowledge hiding of top managers could trigger a trickle-down effect, referring to the fact that the behaviors of the top leaders will affect employees in the formal vertical power chain, given that knowledge hiding can be a multi-participant phenomenon. Therefore, future research could examine the contagious effects of knowledge hiding (e.g., B seeks knowledge from A, but A hides knowledge from B; B then feels lost and hides knowledge from other colleagues), diffusion effects (e.g., B seeks knowledge from A while A hides knowledge from B; A asks C to hide knowledge from B as well), bystander effects (e.g., B seeks knowledge from A, while A hides knowledge from B; C witnesses A's knowledge hiding and is influenced by it, so C also hides knowledge from B and other colleagues), and collective knowledge hiding.
(4) Future scholars should innovate theoretical perspectives and integrate multidisciplinary theories into knowledge hiding research. At present, knowledge hiding research is based mainly on theories such as social exchange, social cognition, social capital, social learning, conservation of resources, territoriality, and psychological ownership. To enrich the field research, it is necessary to diversify the theories. For example, future studies could explore the influence of social exchange relations (e.g., relative LMX) on knowledge hiding, comparing the influence of social LMX and economic LMX on employee willingness to hide knowledge. Future scholars could also conduct multi-interdisciplinary research studies. The research on how an individual's previous workplace behavior affects his or her subsequent workplace behavior has attracted great interest from scholars and mainstream journals in organizational behavior in recent years. Given that knowledge hiding is a typical morality-related behavior, future research could introduce novel and original theoretical viewpoints. For example, a moral balance model and a moral cleansing effect in disciplines such as moral psychology and cognitive psychology, can be used to explore how an individual's previous knowledge hiding behavior influences subsequent behavior in the workplace. Furthermore, knowledge hiding is considered as an emotion-driven behavior. Therefore, scholars could consider employing Lazarus's cognitive–motivational–relational (CMR) theory of emotion (Lazarus, 1991) to better understand the psychological process behind knowledge hiding. Moreover, there is a lack of research on the relationship between individual affect/emotion and knowledge hiding. Therefore, scholars could employ theories, such as affective events theory and self-conscious moral emotion theory, to analyze the subsequent behavior of the hiders and seekers who are driven by affect/emotion.
(5) Research designs need more diversification. Most of the prior studies focus on the individuals, and few research studies focus on both individual and team effects. Knowledge hiding is a complex organizational behavior that concerns individual, team/interpersonal, and organizational levels. Therefore, future research could introduce data tracking technologies, such as big data analysis, to study and compare the dynamic and static (long-term and short-term) effects of multilevel knowledge hiding. Moreover, it is necessary to diversify research methods in the field. Most existing research uses one-wave or multistage surveys, employee self-evaluation, and empirical tests, with few studies using case studies and interviews. These research methods may suffer from a lack of reliability of data sources. Future research could integrate multiple methodologies (e.g., combining case studies, experimental research, surveys, and objective data mining) to verify data, which could improve the internal and external validity of the research and enhance the robustness of conclusions. In particular, it is necessary to focus on the combination of experimental and empirical research, making full use of the strengths of each method to validate the research. Researchers could carry out preliminary tests on relevant hypotheses through experimental research and then supplement them with surveys for secondary verification.
(6) Future research should integrate more cultural, sectoral, and organizational factors to enrich the findings. As discussed in the findings, most of the knowledge hiding data were collected in China and Pakistan. It is necessary to develop the diversity of knowledge hiding data in terms of country of origin. In addition, there is a lack of cross-country academic collaboration. Collaborating across borders could help to generate new ideas and allow for collecting data from different sources. Meanwhile, it would be very interesting to promote cross-country studies to identify the different definitions, perceptions, implementations, and patterns of knowledge hiding, whilst paying more attention to the relationship between cultural dimensions and knowledge hiding. Apart from cross-cultural and cross-country variables, future research could also investigate industry characteristics (such as knowledge-intensive and non-knowledge-intensive industries and masculine and feminine industries), team standards/norms (such as team moral norms), and firm size (small medium enterprises vs. multinational companies) so as to identify the boundary conditions of individual knowledge hiding behavior. Through conducting sector-specific and cross-sector comparison for knowledge hiding, we would be able to adjust knowledge management methods.

## Conclusions

This article provides a systematic review of knowledge hiding. It contributes to the identification of publication patterns on knowledge hiding between 2012 and 2020. Further, we have highlighted the most influential studies, mapped the research gaps, and provided the potential research directions in the field.

This study is not without limitations. We use SCI and SSCI web of science as the databases. Using this literature search method excludes book chapters, reports, unpublished dissertations, with/without peer reviewed conference proceedings, newsletters, government documents, and working papers. Consequently, this review may not have captured the full range of scholarly literature on knowledge hiding. In the future, to reduce the publication bias (Kepes et al., 2012), it would be interesting to include other databases to search literatures, for instance, the work published in the Emerging Sources Citation Index (ESCI) journals can be considered. Second, the research on knowledge hiding is emerging, and some scholars may argue that it is not yet mature enough to review the research field. In our opinion, it is only with such a complete literature review that a clear picture of knowledge hiding research can be developed so that scholars can better define research problems, innovate the research theories and methods, and enrich the field research with a robust framework.

## Data Availability Statement

The raw data supporting the conclusions of this article will be made available by the authors, without undue reservation.

## Author Contributions

PH, CJ, ZX, and CS designed and supervised the study. PH collected the data. PH and ZX analyzed the data. PH, CJ, and CS wrote the manuscript. All authors contributed equally to this manuscript, reviewed, and approved this manuscript for publication.

## Funding

Funding was provided by Huaqiao University's Academic Project Supported by the Fundamental Research Funds for the Central Universities (20SKGC-QT02) and the National Natural Science Foundation of China (72172048).

## Conflict of Interest

The authors declare that the research was conducted in the absence of any commercial or financial relationships that could be construed as a potential conflict of interest.

## Publisher's Note

All claims expressed in this article are solely those of the authors and do not necessarily represent those of their affiliated organizations, or those of the publisher, the editors and the reviewers. Any product that may be evaluated in this article, or claim that may be made by its manufacturer, is not guaranteed or endorsed by the publisher.
